# 807 The Effects of Fish Skin on Wound Healing in a Standardized Pre-clinical Model

**DOI:** 10.1093/jbcr/irae036.347

**Published:** 2024-04-17

**Authors:** Anna-Lisa Pignet, Elisabeth Hofmann, David Hahn, Julia Fink, Andrzej Hecker, Manuel Prevedel, Johanna Einsiedler, Martina Carnieletto, Lars-Peter Kamolz, Petra Kotzbeck

**Affiliations:** Medical University Graz, graz, Steiermark; Joanneum Research Forschungsgesellschaft mbH, COREMED, Graz, Steiermark; Joanneum Research - COREMED, Graz, Steiermark; Joanneum Research & Medical University of Graz, Graz, Steiermark; Medical University Graz, graz, Steiermark; Joanneum Research Forschungsgesellschaft mbH, COREMED, Graz, Steiermark; Joanneum Research - COREMED, Graz, Steiermark; Joanneum Research & Medical University of Graz, Graz, Steiermark; Medical University Graz, graz, Steiermark; Joanneum Research Forschungsgesellschaft mbH, COREMED, Graz, Steiermark; Joanneum Research - COREMED, Graz, Steiermark; Joanneum Research & Medical University of Graz, Graz, Steiermark; Medical University Graz, graz, Steiermark; Joanneum Research Forschungsgesellschaft mbH, COREMED, Graz, Steiermark; Joanneum Research - COREMED, Graz, Steiermark; Joanneum Research & Medical University of Graz, Graz, Steiermark; Medical University Graz, graz, Steiermark; Joanneum Research Forschungsgesellschaft mbH, COREMED, Graz, Steiermark; Joanneum Research - COREMED, Graz, Steiermark; Joanneum Research & Medical University of Graz, Graz, Steiermark; Medical University Graz, graz, Steiermark; Joanneum Research Forschungsgesellschaft mbH, COREMED, Graz, Steiermark; Joanneum Research - COREMED, Graz, Steiermark; Joanneum Research & Medical University of Graz, Graz, Steiermark; Medical University Graz, graz, Steiermark; Joanneum Research Forschungsgesellschaft mbH, COREMED, Graz, Steiermark; Joanneum Research - COREMED, Graz, Steiermark; Joanneum Research & Medical University of Graz, Graz, Steiermark; Medical University Graz, graz, Steiermark; Joanneum Research Forschungsgesellschaft mbH, COREMED, Graz, Steiermark; Joanneum Research - COREMED, Graz, Steiermark; Joanneum Research & Medical University of Graz, Graz, Steiermark; Medical University Graz, graz, Steiermark; Joanneum Research Forschungsgesellschaft mbH, COREMED, Graz, Steiermark; Joanneum Research - COREMED, Graz, Steiermark; Joanneum Research & Medical University of Graz, Graz, Steiermark; Medical University Graz, graz, Steiermark; Joanneum Research Forschungsgesellschaft mbH, COREMED, Graz, Steiermark; Joanneum Research - COREMED, Graz, Steiermark; Joanneum Research & Medical University of Graz, Graz, Steiermark

## Abstract

**Introduction:**

Acellular fish skin grafts (FSG) have proven to be effective in acute and chronic wound healing, as well as in burns. However, the underlying mechanisms promoting healing are still unknown. The aim of this preclincal study was to investigate the effects of fish skin grafts on wound healing properties under standardized conditions in a full-thickness skin defect pig model.

**Methods:**

FSGs were tested in a full-thickness skin defect (3 cm x 3 cm) pig model (n=9, male landrace pigs) and compared intra-individually to untreated control wounds. The experiment lasted for 21 days. Reapplication of the dressings was carried out after 9 days post wounding. Wound scoring, photo-documentation and non-invasive imaging were carried out 5, 9, 14 and 21 days post wounding. Tissue biopsies were sampled at the same time points and subjected to histologic (HE and Masson’s trichrome stains) and gene expression analysis of various cytokines.

**Results:**

As soon as on day 5, wounds treated with FSG showed a thicker formation of new tissue compared to control wounds. Planiometry revealed lower residual wound areas of the wounds treated with FSG, especially on day 9 and 14 post wounding. Gene expression analysis revealed a higher upregulation of the pro (eg. IL-8) and anti-inflammatory markers (eg. IL-10) in the first days after wounding compared to the controls. In the later phases, there were no differences between the two groups. On day 21, control wounds showed severe signs of contraction, which were not observed in the wounds treated with FSG.

**Conclusions:**

FSGs modulate the local immune response by upregulation of both, pro- and anti-inflammatory markers. Moreover, FSGs support wound healing due to the accelerated formation of new tissue and the prevention of wound contraction in a pre-clinical full thickness wound model.

**Applicability of Research to Practice:**

The study gives insights into the mode of action of FSG and therefore aids at redefining the ideal clinical indications for the use of the product.

